# Quantifying the Twitter Influence of Third Party Commercial Entities versus Healthcare Providers in Thirteen Medical Conferences from 2011 – 2013

**DOI:** 10.1371/journal.pone.0162376

**Published:** 2016-09-26

**Authors:** Tejas Desai, Vibhu Dhingra, Afreen Shariff, Aabid Shariff, Edgar Lerma, Parteek Singla, Swapnil Kachare, Zoheb Syed, Deeba Minhas, Ryan Madanick, Xiangming Fang

**Affiliations:** 1 Division of Nephrology, W.G. (Bill) VA Medical Center, Salisbury, North Carolina, United States of America; 2 NOD Analytics, Charlotte, North Carolina, United States of America; 3 Department of Internal Medicine, East Carolina University – Brody School of Medicine, Greenville, North Carolina, United States of America; 4 Division of Endocrinology, Duke University, Durham, North Carolina, USA; 5 Monsanto Company, Raleigh, North Carolina, United States of America; 6 Division of Nephrology, University of Illinois at Chicago College of Medicine, Chicago, Illinois, United States of America; 7 Department of Internal Medicine, Barnes Jewish Hospital, St. Louis, Missouri, United States of America; 8 Department of Surgery, East Carolina University – Brody School of Medicine, Greenville, North Carolina, United States of America; 9 College of Arts and Sciences, College of William and Mary, Yorktown, Virginia, United States of America; 10 Division of Rheumatology, Cedars-Sinai Medical Center, Los Angeles, California, United States of America; 11 Division of Gastroenterology, University of North Carolina, Chapel Hill, North Carolina, United States of America; 12 Department of Biostatistics, East Carolina University – Brody School of Medicine, Greenville, North Carolina, United States of America; Lancaster University, UNITED KINGDOM

## Abstract

**Introduction:**

Twitter channels are increasingly popular at medical conferences. Many groups, including healthcare providers and third party entities (e.g., pharmaceutical or medical device companies) use these channels to communicate with one another. These channels are unregulated and can allow third party commercial entities to exert an equal or greater amount of Twitter influence than healthcare providers. Third parties can use this influence to promote their products or services instead of sharing unbiased, evidence-based information. In this investigation we quantified the Twitter influence that third party commercial entities had in 13 major medical conferences.

**Methods:**

We analyzed tweets contained in the official Twitter hashtags of thirteen medical conferences from 2011 to 2013. We placed tweet authors into one of four categories based on their account profile: healthcare provider, third party commercial entity, none of the above and unknown. We measured Twitter activity by the number of tweet authors per category and the tweet-to-author ratio by category. We measured Twitter influence by the PageRank of tweet authors by category.

**Results:**

We analyzed 51159 tweets authored by 8778 Twitter account holders in 13 conferences that were sponsored by 5 medical societies. A quarter of all authors identified themselves as healthcare providers, while only 18% could be identified as third party commercial entities. Healthcare providers had a greater tweet-to-author ratio than their third party commercial entity counterparts (8.98 versus 6.93 tweets). Despite having less authors and composing less tweets, third party commercial entities had a statistically similar PageRank as healthcare providers (0.761 versus 0.797).

**Conclusion:**

The Twitter influence of third party commercial entities (PageRank) is similar to that of healthcare providers. This finding is interesting because the number of tweets and third party commercial entity authors required to achieve this PageRank is far fewer than that needed by healthcare providers. Without safety mechanisms in place, the Twitter channels of medical conferences can devolve into a venue for the spread of biased information rather than evidence-based medical knowledge that is expected at live conferences. Continuing to measure the Twitter influence that third parties exert can help conference organizers develop reasonable guidelines for Twitter channel activity.

## Introduction

Medical conference organizers must strike a balance with commercial entities (e.g., pharmaceutical companies and device manufacturers). Third parties are needed to offset the cost of many national scientific meetings and provide valuable information about the latest developments in the field [1,2]. Concurrently, conference organizers must mitigate “detailing”: the process in which third parties have direct and unregulated access to conference attendees (learners) [3,4]. Organizers have reached this balance in live conferences by: 1) not allowing third parties to select speakers at plenary and other sessions, 2) not allowing third parties to pass out literature in-and-around classrooms, and 3) restricting learner access to third parties to one geographic location ("exhibition hall") and only during specific periods of time that do not conflict with other scientific sessions [2]. Theoretically, these safety mechanisms allow a learner to experience a live medical conference without ever exposing him/herself to a third party.

This model has not been replicated in the increasingly popular Twitter hashtag channels (channels) [5,6]. Twitter channels are open and freely accessible online Twitter streams that allow learners, conference organizers, and third parties to share information with each other [5,6]. These channels are not covert; conference organizers encourage attendees to use these channels through official publications and signage at the conference itself. In part because they are freely available to the public, Twitter channels enhance live conferences and an increasing number of medical conferences are incorporating them into their annual meetings [7–18]. Although promoted by conference organizers, the medical conference Twitter channels are unregulated; this allows third parties direct access to learners that they cannot achieve at a live conference. Detailing on Twitter exposes learners to third parties and facilitates the transfer of biased information in an environment that does not have established safety mechanisms in place [1,3,4]. Theoretically, third parties can exert a greater influence over learners through Twitter detailing.

In this investigation, we used established methods to explore the Twitter influence that third parties have in the Twitter channels of thirteen prominent medical conferences from 2011–2013. In the unregulated realm of Twitter channels, we hypothesized that third parties have a comparable Twitter influence score as any other group.

## Methods

### Data Set

We identified five (5) medical societies that promoted the Twitter channels of their respective annual meetings. These societies assigned a conference-specific hashtag for each channel and registered each with the Healthcare Hashtag Project (HHP; symplur.com). We queried the HHP database for all conference-specific tweets using the pre-assigned hashtags. Table 1 shows the thirteen conferences that were included in the data set. We collected a) date and time of tweet, b) Twitter username of the tweet author, c) content of the tweet, and d) Twitter username(s) of individuals/organizations mentioned within the body of a tweet (@mentions).

**Table 1:** Baseline Data.

Medical Society / Conference Organizer(s)	Dates	Hashtag	Healthcare Hashtag Project URL (shortened)	Tweets (No.)
*Conference Name*				
American College of Cardiology*				8505
*2012 Annual Meeting*	*3/24/2012–3/27/2012*	*#acc12*	http://goo.gl/Os3f0C	*2951*
*2013 Annual Meeting*	*3/9/2013–3/11/2013*	*#acc13*	http://goo.gl/nnwEPK	*5554*
American Society of Nephrology				4299
*Kidney Week 2011*	*11/8/2011–11/13/2011*	*#kidneywk11*	http://goo.gl/m3UvNm	*583*
*Kidney Week 2012*	*10/30/2012–11/4/2012*	*#kidneywk12*	http://goo.gl/ni5CC6	*1137*
*Kidney Week 2013*	*11/5/2013–11/10/2013*	*#kidneywk13*	http://goo.gl/8vog3n	*2579*
American Society of Clinical Oncology				31991
*2011 Annual Meeting*	*6/3/2011–6/7/2011*	*#asco11*	http://goo.gl/GX3svS	*7531*
*2012 Annual Meeting*	*6/1/2012–6/5/2012*	*#asco12*	http://goo.gl/HIky9X	*9555*
*2013 Annual Meeting*	*5/31/2013–6/4/2013*	*#asco13*	http://goo.gl/IOS4pf	*14905*
American Gastroenterological Association and American Society for Gastrointestinal Endoscopy and American Association for the Study of Liver Diseases and The Society for the Surgery of the Alimentary Tract				5123
*Digestive Disease Week 2011*	*5/7/2011–5/10/2011*	*#ddw11*	http://goo.gl/xpwZpY	*1199*
*Digestive Disease Week 2012*	*5/19/2012–5/22/2012*	*#ddw12*	http://goo.gl/fgM3di	*1720*
*Digestive Disease Week 2013*	*5/18/2013–5/21/2013*	*#ddw13*	http://goo.gl/QmL66C	*2204*
American Academy of Dermatology*				1241
*2012 Annual Meeting*	*3/16/2012–3/20/2012*	*#aad12*	http://goo.gl/ZrcGOj	*585*
*2013 Annual Meeting*	*3/1/2013–3/5/2013*	*#aad13*	http://goo.gl/BeCl23	*656*

*Conference Twitter channel for the 2011 meeting was not registered with the HHS & unavailable for analysis

### Content Analyses

We performed five (5) separate analyses to identify the types of content within our data set. First we categorized each tweet as an “original” or “retweet”. We defined “original tweets” as messages composed by the same author who tweeted the message. We defined “retweet” as a message composed by a different author than the one who tweeted the message. Realizing that many medical conferences expose attendees to products/services offered by third party commercial entities, we defined “advertisements” as those tweets that were soliciting the attendees to a) use a product or service or b) visit an exhibition booth to learn more about a product/service. Moreover many of these products/services are offered by commercial entities that are publicly traded corporations. Thus we defined “financial tweets” as those messages whose content contained information about the underlying financial security (stock) of a particular commercial entity. In our fourth analysis we identified tweets that contained hyperlinks (URLs) to multimedia files (pictures or videos or both); we defined such tweets as “enhanced”. Finally, we calculated word frequencies in the remaining tweets that were not categorized as “advertisements” or “financial” in order to identify the most popular scientific topics that were discussed within each medical discipline. Table 2 provides representative examples of tweets that satisfied each category definition.

**Table 2:** Categories and examples of Tweets based on content.

Characteristic	Representative Example
Original	Packed Cochrane IBD symposium: what have the past 20 years taught us? #ddw13
Retweet	RT @ClevelandClinic: 17% of children in the U.S are obese and at risk for serious health complications #DDW13 #childhoodobesity
Advertisement	50% of newly diagnosed #myeloma patients present with #renal insufficiency. Learn more at our Booth 1404 #kidneywk13 http://t.co/n0ogvIyAos
Financial Security	Monthly shot of Amgen PCSK9 drug cuts bad cholesterol up to 66%; Regeneron's PCSK9 data due today at #ACC12 http://t.co/JfRhhHcr $AMGN $REGN
URL	High frequency of #mutations seen in black women with #breastcancer. @ASCO #NGS #genes #ASCO13 http://t.co/ZT0eJgk6rj

### Categorization of Account Holders

In order to quantify the influence that third parties exert in Twitter channels, we categorized every tweet author/account holder mentioned within the body of a tweet (@mentions) into one of four categories: a) healthcare provider (HCP), b) third party commercial entity (third party), c) unclear identity, and d) none of the above. Table 3 defines each category and provides a representative example. We used each account holder’s Twitter profile to ascertain under which category that account should be. Categorization was done from January to April 2014. We did not perform additional Internet searches (e.g., Facebook or Google search) of accounts categorized as “unclear”. None of the investigators contacted any of the account holders to determine their identity (see Discussion for further information).

**Table 3:** User categories and examples.

Category	Definition	Representative Example	Twitter Profile
Healthcare Provider	Individual or organization whose primary purpose is to disseminate medical information or provide clinical care for patients	@nephondemand	Tejas Desai, MD. Creator of Nephrology On-Demand & Kidney Konnection & Nephrology Fellowship Director @ ECU. I conduct research in social media & medicine & program iOS Apps
3rd Party Commercial Entity	Organization or individual representing an organization whose primary purpose is to provide a product or service to medical professionals and/or patients	@mmsholdings	MMS Holdings Inc. MMS Holdings Inc. is a global niche pharmaceutical service organization that focuses on regulatory submission support for the pharma and biotech industries.
None of the Above	Individuals or organizations that are unrelated to healthcare or the purpose of the scientific meeting	@RdgTerminalMkt	The Reading Terminal Market—Since 1893
Unclear Identity	Individual or organizations whose Twitter profile was vague or empty	@KhaliqWhy	Khaliq. Seeking Knowledge

To ensure inter-rater reliability when categorizing Twitter account holders, we performed a Light’s kappa statistic on a different set of previously published Twitter data [19]. The Light’s kappa score was 0.72 for eight raters (AS, AS, VD, PS, SK, ZS, EL, DM).

### Defining and Measuring Twitter Activity

We assessed Twitter activity using two methods. In the first method, we measured the number of distinct account holders per category that authored at least one tweet in one of the 13 conferences analyzed. We defined a high Twitter activity as that category with the largest number of account holders.

In the second method, we measured the total number of tweets authored by account holders in each category. We calculated the tweet:author ratio by dividing the number of tweets composed by the total number of authors within a particular category. We ascribed the greatest Twitter activity to that category with the highest ratio.

### Defining and Measuring Twitter Influence

Keller, Leavitt et al, and Antoniadis et al defined influence as one’s ability (user A) to affect the behavior of another (user B) [20–22]. On Twitter, that behavior is represented by the actions that user B takes after reading user A’s tweet. Twitter allows user B to take one of two actions: replying or retweeting a message [20,22]. In its most primitive form, Twitter influence is a quantification of the number of replies or retweets that user A accrues [20,22]. The greater the sum of replies to and retweets of user A’s tweets, the more influential user A is on Twitter. Although many social media research investigations (including this investigation) use this definition of Twitter influence, they do not use the arithmetic sum to quantify it. Among the reasons for disuse is that the sum of replies and retweets ignores the existing level of Twitter influence with which a user begins. To account for the existing level of Twitter influence one has, and to be consistent with prior investigations that quantify Twitter influence, we measured influence using the PageRank [20,22–34].

We calculated the PageRank of every account holder that was mentioned (@mentions) in the body of a tweet. The @mentions include both the replies to and retweets of one’s original tweet. Originally developed by Page, Brin, Motwani and Winograd, the PageRank is a link-based algorithm and considered by Williams, Baldwin, and Rubel to be the best measure of social media influence [23–26]. As described by Abdullah, in the PageRank “a link from a page to another page is understood as a recommendation and the status of the recommender is important” [27]. A webpage, to which many others are linked, is considered an influential webpage and is given a high PageRank [23,27]. Its PageRank increases even more when the linking webpages are influential as well (i.e., have their own high PageRanks) [23,27–29]. Similarly, a Twitter account that is mentioned (@mentions) many times and/or mentioned by other influential Twitter accounts will, itself, appropriately receive a high PageRank [31]. Indeed a number of investigators, including Abdullah, Kwak et al and Bakshy et al, have successfully used the PageRank to accurately measure Twitter influence using @mentions [26,27,30–33]. The PageRank of @mentions is also known as the “Influence Index” and is used by the independent research firm Twitalyzer to measure one’s Twitter influence [34]. It is also the preferred method of measuring Twitter influence by Evan Williams, co-founder of Twitter [34].

### Privacy Considerations for Account Holders

The tweets collected from the HHP contained identifying information or links to such information. The same identifying information is freely available to the general public through the Library of Congress [35,36]. Twitter’s Terms and Conditions warn account holders of the public nature of tweets, specifically, “what you say on Twitter may be viewed all around the world instantly” [12]. Perhaps because such identifying information is freely accessible, prior investigators have not requested approval from their local institutional review boards [13,26,27,30–33]. Currently there are no expectations for researchers to gain approval from any external agency (government, Twitter, or others) to research Twitter data [37]. In many investigations, including our own, researchers have adopted the “distance principle”, explained by Buchanan et al [38]. Given that our investigation was an observation of data in the public space and did not involve direct interaction with any account holder, the “distance principle”, along with the precedent set forth by previous investigators, supported our belief that external committee review was unwarranted [37–40].

Nevertheless, the identifying information within each tweet was as critical to our investigation as our ethical use of it. Therefore, we designed our methods in accordance with the United States Department of Homeland Security’s 2012 Menlo Report–a guide for investigators performing “communication technology research” [41]. We also designed our methods to conform to the British Psychological Society’s guidelines for “Internet-mediated research” [42]. Finally, we complied with the six ethics guidelines recommended by Rivers and Lewis when analyzing “big data” [37]. Our adherence to these strict and established guidelines satisfied our professional sense of duty/ethics to maintain the privacy of the account holders whose Twitter activities comprised our data set.

### Statistical Considerations

We performed content analyses using NOD Analytics (goo.gl/mfziXG). We used WordItOut (worditout.com) to graphically represent the popular scientific topics in each Twitter channel. We calculated frequencies per category for: 1) number of Twitter accounts that authored tweets, 2) number of Twitter accounts that were mentioned within a tweet, 3) number of tweets composed. We performed chi-square tests to compare these data using JMP Pro version 10.0.0 (SAS, Cary, North Carolina). We calculated PageRank using the NodeXL plugin (nodexl.codeplex.com) for Microsoft Excel 2013 (Microsoft, Redmond, Washington). Median and interquartile ranges for the PageRank were calculated and compared using the Kruskal-Wallis test. Each group needed to have at least 8671 @mentions in order to have achieved an 80% power to detect a 0.2 difference in PageRank. To mitigate any future concern about the lack of reproducibility of our results, we 1) did not perform subgroup analyses of Twitter influence by conference and 2) followed recent guidelines that make “classical hypothesis testing more congruent with evidence thresholds for Bayesian tests” [43]. As a result, the significance level was set at p < 0.005 [43].

This investigation conforms to STROBE guidelines for observational research and SAMPL guidelines for statistical reporting [44,45].

## Results

### Baseline Data

We collected 51159 tweets, authored by 8778 Twitter account holders, in 13 conferences, sponsored by 5 medical societies, from 2011 to 2013 (Table 1). Our data set represents 94.6% of tweets and 78.1% of authors in the HHP. The remaining data was either lost during the extraction process from the HHP or could not be parsed correctly by the software we used. The largest number of tweets and authors was in the 2013 American Society of Clinical Oncology’s annual meeting (15120 and 3156, respectively).

### Content Analyses

**Fig 1:**
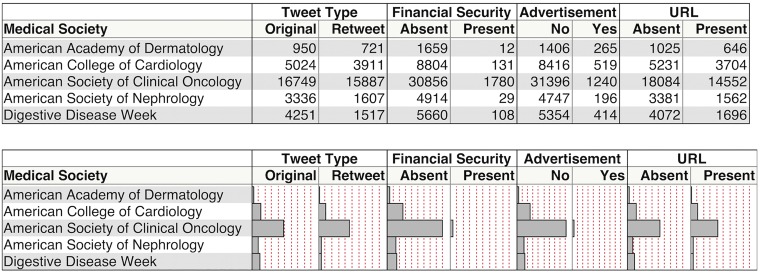
Content Analyses.

In every Twitter channel there were more original tweets composed (N = 30310) than retweets generated (N = 23643). The overall original tweet:retweet ratio was 1.28:1: the lowest ratio belonging to the American Society of Oncology’s annual meetings and the highest to the Digestive Disease Week meetings (1.05 versus 2.80, respectively). There were very few advertisements: the non-advertisement:advertisement ratio in our data set was 19.48:1 (Fig 1). A similar favorable ratio was seen amongst tweets containing financial information. Finally, many more tweets were exclusively textual than associated with a multimedia image: a ratio 1.43:1.

Unsurprisingly the most common scientific topic in the entire data set revolved around the “patient” (e.g., patient care, patient centered, patient specific, etc.). Popular topics in the American College of Cardiology Twitter channels were three clinical trials and three medications. The clinical trials were: CORONARY (sponsored by Canadian Institute of Health Research), PREVAIL (sponsored by Boston Scientific Corporation) and HPS2-THRIVE (sponsored by Merck and Company, Inc.). The popular medications were: Cangrelor (by The Medicines Company), Niacin (by AbbVie, Inc.), and Rivaroxaban (by Janssen Pharmaceuticals). Popular topics in the American Society of Oncology Twitter channels were specific diseases (melanoma, NSCLC [non-small cell lung cancer], myeloma, and breast cancer). We did not identify specific medications or clinical trials as popular topics in #asco11, #asco12, and #asco13.

**Fig 2:**
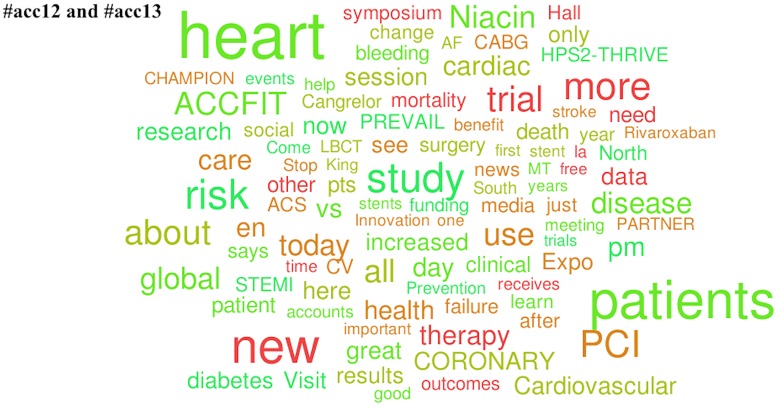
Popular topics at the 2012 and 2013 American College of Cardiology Annual Meetings. Word clouds exclude prepositions, conjunctions, articles, numbers, Twitter usernames, and official conference-specific hashtags.

**Fig 3:**
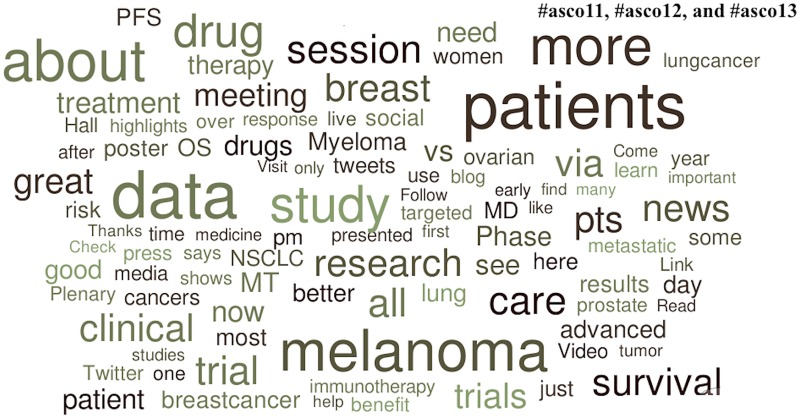
Popular topics at the 2011, 2012, and 2013 American Society of Clinical Oncology Annual Meetings. Word clouds exclude prepositions, conjunctions, articles, numbers, Twitter usernames, and official conference-specific hashtags.

**Fig 4:**
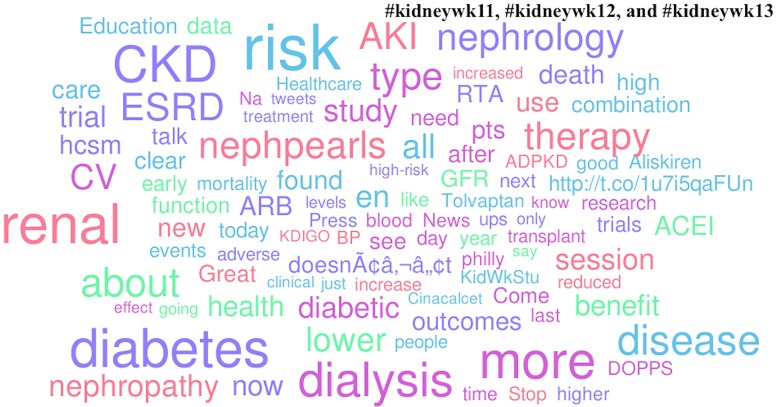
Popular topics at the 2011, 2012, and 2013 American Society of Nephrology Annual Meetings. Word clouds exclude prepositions, conjunctions, articles, numbers, Twitter usernames, and official conference-specific hashtags.

**Fig 5:**
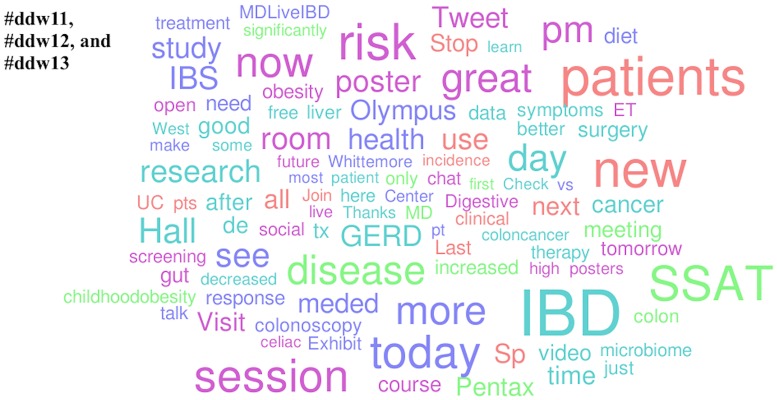
Popular topics at the 2011, 2012, and 2013 Digestive Diseases Week. Word clouds exclude prepositions, conjunctions, articles, numbers, Twitter usernames, and official conference-specific hashtags.

**Fig 6:**
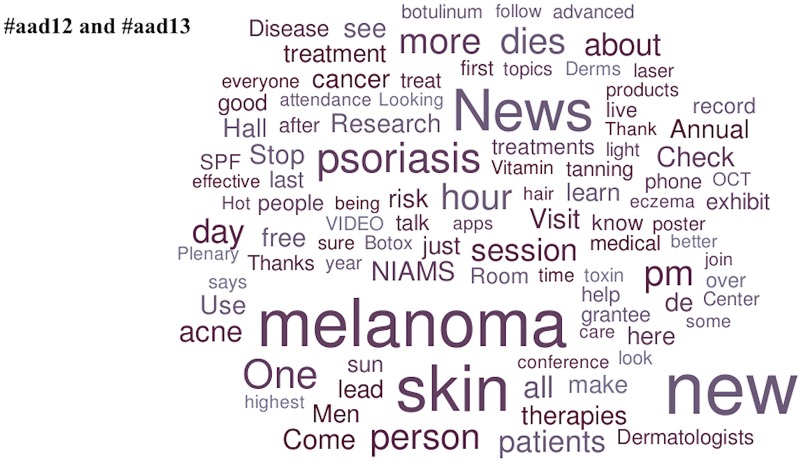
Popular topics at the 2012 and 2013 American Academy of Dermatology Annual Meetings. Word clouds exclude prepositions, conjunctions, articles, numbers, Twitter usernames, and official conference-specific hashtags.

Medications and diseases were the popular topics in the American Society of Nephrology Twitter channels. Aliskiren (by Novartis Pharmaceuticals), tolvaptan (by Otsuka Pharmaceutical Company, Ltd.), and cincalcet (by Amgen, Inc.) competed with CKD (chronic kidney disease), AKI (acute kidney injury), and ESRD/dialysis for popularity. In the Twitter channels of Digestive Disease week, the popular diseases discussed were IBD (inflammatory bowel disease), IBS (irritable bowel syndrome) and GERD (gastroesophageal reflux disease). Uniquely popular in the #ddw11, #ddw12, and #ddw13 channels were explicit mentions of third party commercial entities (specifically Pentax Medical and Olympus America Medical). Lastly, the only appearance of a governmental agency amongst the list of popular topics was in the #aad12 and #aad13 channels (NIAMS [National Institute of Arthritis and Musculoskeletal Skin Diseases]) (Figs 2–6).

### Twitter Activity and Influence

Nearly 61% of the authors had a Twitter profile that identified them (Table 4). In this group, there were 2173 (25%) healthcare providers and 1575 (18%) third party entities (p < 0.0001). The largest group of authors could not be identified (3412; 39%; p < 0.0001).

**Table 4:** Measures of Twitter Activity and Influence.

**TWITTER ACTIVITY**	**Third Party Commercial Entity**	**Healthcare Provider**	**None of the Above**	**Unclear Identity**
Total Authors	1575	2173	1617	3413
Total Tweets	10916	19503	8105	12635
Tweets:Author Ratio	6.931	8.975	5.012	3.702
**TWITTER ENGAGEMENT**	**Third Party Commercial Entity**	**Healthcare Provider**	**None of the Above**	**Unclear Identity**
Unique @mentions	683	864	772	997
Total @mentions	7834	18341	8011	5811
**PAGERANK**				
*10th Percentile*	*0*.*313*	*0*.*316*	*0*.*304*	*0*.*297*
*25th Percentile*	*0*.*425*	*0*.*441*	*0*.*405*	*0*.*401*
*Median*	*0*.*761*	*0*.*797*	*0*.*677*	*0*.*591*
*75th Percentile*	*1*.*391*	*1*.*546*	*1*.*124*	*1*.*005*
*90th Percentile*	*3*.*103*	*3*.*089*	*2*.*158*	*1*.*921*

Despite being the greatest number of authors, those with unclear identities did not compose the greatest number of tweets (Table 4). The tweet:author ratio for unidentified Twitter account holders was only 3.7. Healthcare providers composed 19503 tweets and had a tweet:author ratio greater than that of third party entities (8.98 versus 6.93 tweets per author; p < 0.0001).

In our data set, a total of 3316 Twitter accounts were mentioned a total of 39997 times (Table 4). Healthcare providers were mentioned nearly 46% of the time, while third party commercial entities were mentioned less than 20% of the time. The sum total of @mentions in the healthcare provider and third party categories was 26175: 1.5 times greater than the 17341 @mentions needed to achieve 80% power. The median PageRank for healthcare providers was the highest amongst the four categories. However, there was no statistical difference between it and the median PageRank for third party commercial entities (0.797 versus 0.761, respectively; p 0.175).

## Discussion

### Third Party Twitter Influence

Third party commercial entities had a statistically similar PageRank as healthcare providers (0.761 versus 0.797, respectively) despite having significantly fewer authors (1575 versus 2173, respectively) and significantly less Twitter activity (6.931 versus 8.975 tweets/author, respectively). This suggests that third parties are equally influential in the Twitter channels of scientific meetings as healthcare providers; a parity that is difficult to achieve at live conferences. Admittedly, there are no investigations that measure third party influence at live conferences. Perhaps the lack of data is due to conference organizers’ financial reliance on third parties to sponsor their conferences. In 2009, third parties gave close to $850 million dollars of sponsorships to various medical conferences [1]. In 2011, 75% of conference organizers received third party financial support [2]. Third parties provide printed and digital conference materials, travel grants, and meals gratis. This financial dependence may preclude any scientific study of third party influence at live conferences. Nevertheless, conference organizers mitigate third party influence by geographically isolating third parties, curtailing their “hours of operation”, and independently selecting topics and speakers for the conference agenda [2].

Conference organizers do not depend on the financial support of third parties to maintain active Twitter channels. Creating and registering a conference-specific hashtag and composing tweets are free. Yet not one of the eight conference organizers (in any of the 13 conferences studied) implemented any safeguards to limit third party “detailing” [3]. As a former third party representative, Ahari outlined eight forms of detailing used by third parties to influence individuals [3]. All eight can be easily adapted to work in Twitter channels. Indeed any message from a third party is more likely to place a favorable bias on that party’s product/service than unprejudiced evidence-based medicine [1].

Jalali, Wood, and others have suggested that conference organizers learn how their respective Twitter channels are being used/misused in order to curtail the Twitter influence that third parties have within them [16,31,44,46]. Our study is the first to elucidate this use/misuse by various groups. Second, more must be done to establish guidelines for third party activities in Twitter channels. There are plenty of well-intentioned recommendations on the use of Twitter by healthcare providers and conference organizers [17,47]. There are no comparable recommendations for third parties or their interactions with HCPs or conference organizers [48]. Both the Pew Charitable Trusts and American Medical Student Association discuss how conference organizers can mitigate conflicts of interest (COI), but neither offer specific guidelines in managing COIs within social media streams [1,49,50]. Therefore, the investigators of this study recommend the following to bring the medical community closer to such guidelines:

- Conference organizers should publicly state in their Twitter channel that third party entities should declare themselves as such in their respective Twitter profiles [36]
- Conference organizers should insist that third parties compose tweets that disseminate scientific facts and not solicitations for products/services
- If third parties wish to solicit for a product/service, they should include an additional hashtag in the body of their tweet (e.g., #ad) to allow participants within the channel to filter out such tweets
- Conference organizers should encourage third parties to restrict their Twitter activity to coincide with their live “hours of operation”
- Conference organizers should task independent individuals/groups to annually measure the PageRanks for each Twitter account mentioned (@mentions) within their conference-specific hashtag
- Conference organizers should target third party accounts with abnormally high PageRanks for further education about best-practices within their respective Twitter channel

These recommendations would align third party activities in Twitter channels with their activities at live conferences. These recommendations are not mandates, but rather reasonable suggestions that are neither burdensome to conference organizers nor offensive to third party commercial entities. These recommendations are made with the same intentions as those that guide physician activity on Twitter [17,47]. In both cases, monitoring, rather than enforcement, is a key component of assessing compliance. Measuring yearly PageRank scores, as performed in this investigation, will help all parties monitor compliance. Conference organizers can implement targeted re-education efforts for those third parties that require additional assistance.

### PageRank versus other measures of Twitter Influence

The PageRank of @mentions has been used by a number of Twitter researchers and is considered the closest estimation of Twitter influence [20,22,24–27,30,32,33]. Indeed even commercial research firms, such as SEOmoz and Twitalyzer, use the PageRank of @mentions to measure Twitter influence for their clients [34,41]. As Bray and Peters have indicated, mentioning someone in one’s tweet represents a major commitment to that person [51]. The more a person is mentioned, the more they effect the conversation and the greater the Twitter influence they exert [31,34,51].

A common misperception is that the number of followers or impressions (which equals the product of the number of followers and tweets composed) is an accurate measure Twitter influence. Any metric that uses the number of followers and/or tweets often results in false calculations of Twitter influence [20,22,52]. Bots can artificially inflate the number of tweets composed, causing the impressions to be misleadingly elevated. Moreover, the number of followers or impressions excludes any interaction between participants. Perhaps for these reasons impressions and the number of followers are considered “vanity” metrics: easy to calculate but of little value in measuring one’s Twitter influence [20,22,51].

Twitter researchers do not perform content analyses to measure influence [20,26]. Neither this investigation nor the studies referenced in this report have analyzed tweet content to measure Twitter influence. We have analyzed the content of the tweets in our data set to give the reader, if needed, a contextual framework upon which to interpret our results. Indeed Cha et al has mathematically analyzed various metrics to measure Twitter influence and concluded that the PageRank of @mentions was one of the best ways to do so [32].

### Unclear Identities on Twitter

There were 3413 Twitter accounts that could not be identified because their Twitter profiles were vague or empty. These accounts generated only 24.7% of the total tweets analyzed. We consciously avoided using alternative methods to identify these accounts. In accordance with recommendations by Farnan and McKee, we assumed that account holders with vague profiles wanted to remain anonymous [39,53]. To respect these wishes, we did not contact any author or perform additional Internet searches to ascertain their identities [37,38].

### Strengths

Perhaps the greatest strength of this investigation is its breadth (13 conferences sponsored by 8 medical societies) and depth (51159 tweets). Chaudhry et al conducted an analysis of 12644 tweets from 2 conferences sponsored by one medical society while Jalali et al analyzed 10937 tweets from 4 conferences sponsored by as many medical societies. [13,54]. We measured Twitter influence by calculating the PageRank of @mentions–the recommended metric by a number of researchers, commercial research firms, and the co-founder of Twitter [24–26,30,32,34,41]. We conformed to three well-established sets of guidelines for conducting Internet-based research and respected the privacy of those users who wanted to remain anonymous [1,37–39,41,53]. Finally, and to the best of our ability we have reported our findings in accordance with two sets of research-reporting guidelines [44,45]

### Limitations

As with many studies that study Twitter activity, we were unable to directly measure influence. We used a proxy metric to measure Twitter influence: the PageRank. However, the PageRank is considered one of the best and most commonly used markers of social media influence [20,22,23–27,30–34]. Second, our recommendations are unenforceable. As with any medical guideline, our recommendations are meant to serve as a basis upon which best practices can be developed. The value of recommendations does not emanate from their enforceability, but rather from their ability, over time, to percolate throughout the Twitter medical community and change practice. To-date there is no known evidence that any set of voluntary guidelines regarding Twitter use can or cannot lead to changes in behavior. Third, our data set did not include those Twitter profiles that we could not identify. While 39% of all users were unknown, we met our power threshold. We calculated PageRanks using 39997 @mentions: 4.6 times more than the 8671 @mentions needed to achieve 80% power.

## Conclusion

Using the PageRank as a surrogate marker, third party commercial entities exert an equal Twitter influence as healthcare providers in the Twitter channels of medical conferences. Without safety mechanisms in place, Twitter channels can devolve into a venue for the spread of biased information rather than evidence-based medical knowledge, as seen at live conferences. Continuing to measure the Twitter influence that third parties exert can help conference organizers develop reasonable guidelines for Twitter channel activity.
